# Circ-BPTF promotes bladder cancer progression and recurrence through the miR-31-5p/RAB27A axis

**DOI:** 10.18632/aging.101520

**Published:** 2018-08-09

**Authors:** Junming Bi, Hongwei Liu, Zijian Cai, Wei Dong, Ning Jiang, Meihua Yang, Jian Huang, Tianxin Lin

**Affiliations:** 1 Department of Urology, Sun Yat-Sen Memorial Hospital, Sun Yat-Sen University, Guangzhou PR China; 2 Guangdong Provincial Key Laboratory of Malignant Tumor Epigenetics and Gene Regulation, Sun Yat-Sen Memorial Hospital, Sun Yat-Sen University, Guangzhou PR China; * Equal contribution

**Keywords:** circ-BPTF, miR-31-5p, RAB27A, bladder cancer, prognosis

## Abstract

Circ-BPTF (hsa_circ_0000799) is a novel circular RNA derived from BPTF exons. Although BPTF is a well-studied predecessor gene, the characteristics and functions of circ-BPTF have not yet been reported. Here, we show that expression of circ-BPTF is increased in bladder cancer (BCa) tissues and cell lines compared with noncancerous tissues and cell lines. Consistently, BCa patients with higher expression levels of circ-BPTF were found to have higher tumor grades and poorer prognosis. Functionally, knockdown of circ-BPTF inhibited tumor progression in vitro and in vivo. Mechanistically, a target microRNA of circ-BPTF was confirmed to be miR-31-5p, and miR-31-5p mimics partially reversed the effect of circ-BPTF. Furthermore, RAB27A was predicted and shown to be a target of miR-31-5p, and circ-BPTF attenuated the anti-oncogenic effect of miR-31-5p and consequently enhanced RAB27A expression. In summary, our findings reveal that circ-BPTF promotes BCa progression through the miR-31-5p/RAB27A axis, suggesting that circ-BPTF may be a potential target for BCa treatment.

## Introduction

Bladder cancer (BCa) is one of the most prevalent cancers worldwide, with an estimated incidence of 430,000 cases each year [1]. Despite improvements in multiple therapeutic approaches such as surgery, intravesical chemotherapy and immunotherapy, rates of recurrence and progression within 5 years remain high [2,3]. Accumulating evidence suggests that BCa represents a group of molecularly and clinicopathologically heterogeneous diseases [4]. Therefore, understanding the molecular pathology of BCa is critical for the diagnosis, classification and treatment of this disease.

Circular RNAs (circRNAs), a class of noncoding RNAs ubiquitous in the cytoplasm of various eukaryotic cells, are mainly derived from exonic regions of protein-coding genes [5]. The molecules are cyclized, lacking 5’ caps and 3’ poly (A) tails [6]. Due to their abundance and stability in plasma and tissues, several circRNAs have been considered potential biomarkers for the diagnosis and prognosis of various cancers such as hepatocellular carcinoma (HCC) [7] and gastroenteric tumors [8].

Mechanistically, circRNAs are mainly regarded as microRNA sponges, protecting their target genes from cleavage by certain miRNAs [9]. More importantly, by regulating the cell cycle, signal transduction and transcription, circRNA-miRNA networks closely correlate with the occurrence, progression and prognosis of cancer [10]. For instance, the circRNA cSMARCA5 promotes expression of TIMP3 by sponging miR-17-3p and miR-181b-5p and therefore inhibits the proliferation and migration of HCC cells [7]. In glioblastoma, circNT5E reportedly binds directly to miR-422a to abrogate its oncogenic activity [11]. Some circRNAs have also been found to exhibit their regulatory functions through RNA-binding proteins (RBPs) and can even serve as polypeptide-encoding nucleic acids [12]. Despite the increasing number of studies on circRNAs, there are few reports concerning the biological role of circRNAs in BCa [13–17], which needs to be explored.

circ-BPTF (hsa_circ_0000799) is a novel circRNA that is derived from exons 21 to 27 of the bromodomain PHD finger transcription factor (BPTF) gene via back-splicing. Although this predecessor gene has been investigated for its oncogenic role in lung adenocarcinoma [18] and colon cancer [19], there is little knowledge to date regarding circ-BPTF.

MicroRNAs (miRNAs) comprise a group of RNAs that exert biological functions by binding to the 3’-UTR of target mRNAs. miRNAs are known to have important functions in multiple biological processes, including suppressing or stimulating tumor progression. According to previous studies, miR-31-5p acts as a tumor suppressor in BCa [20]. Specifically, enhanced expression of miR-31-5p increases the sensitivity of BCa to chemotherapy [20], whereas reduced expression is associated with BCa progression and a poor prognosis [21,22]. Nevertheless, the regulatory pathways in which miR-31-5p participates remain unclear.

RAB27A, an oncogene belonging to the Rab family, is involved in protein transport and small GTPase-mediated signal transduction [19]. Members of the Rab family are involved in multiple processes in tumorigenesis [19]. According to previous studies, RAB27A is strongly relevant to the progression of tumors, such as HCC [23], pancreatic cancer [24] and melanoma [25]. In melanoma, RAB27A serves as the target of miR-31-5p [26], and studies have also shown that elevated levels of RAB27A expression promote BCa proliferation and chemoresistance [27]. However, the reason why RAB27A is overexpressed in BCa remains largely unknown.

In the present study, circ-BPTF was found to be significantly overexpressed in BCa, and this circRNA was closely associated with poor prognosis, tumor grade and recurrence in BCa patients. Functional experiments revealed that circ-BPTF promotes BCa progression in vivo and vitro, and such BCa growth and metastasis occurs through the miR-31-5p/RAB27A axis.

## RESULTS

### Characterization of circ-BPTF in BCa

**Figure 1:**
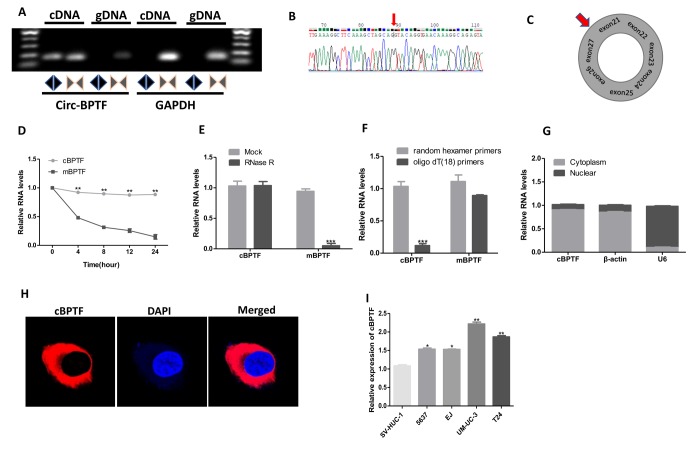
**Validation and characteristics of circ-BPTF in BCa cells. **(**A**) Gel electrophoresis analysis of the PCR products of circ-BPTF and linear BPTF. Divergent primers amplified circ-BPTF only in cDNA, whereas, convergent primers produced linear transcript in both cDNA and gDNA. GAPDH was applied as a linear control. (**B**) Sanger sequencing of circ-BPTF products amplified by PCR. The back-splice junction site was marked by the red arrow. (**C**) Schematic illustration showed that circ-BPTF was cyclized from exon 21 and exon 27 of BPTF. (**D**) The circular and linear form of BPTF levels were examined by qPCR after exposure to Actinomycin D in T24 cells. (**E**) Circ-BPTF and linear BPTF levels were detected by qPCR in T24 cells treated with or without RNase R. (**F**) qPCR analysis of circ-BPTF and linear BPTF using random or oligo dT (18) primers in the reverse transcription process. (**G** and** H**) Circ-BPTF is mainly presented in the cytoplasm of T24 cells verified by nuclear mass separation assay and FISH. (**I**) Expression of circ-BPTF in BCa cell lines (5637, EJ, UM-UC-3, T24) and normal urothelial cells (SV-HUC-1) was detected by qPCR. Data indicate means ± SEM. **P<0.01, ***P<0.001.

To investigate the characteristics of circ-BPTF, qPCR was performed with divergent primers, and agarose gel electrophoresis (Figure 1A) and Sanger sequencing (Figure 1B) on the PCR products were performed. The results indicated that circ-BPTF was expressed in BCa cell lines, confirming the back-splice junction of circ-BPTF. Furthermore, we found circ-BPTF to be derived from exons 21, 22, 23, 24, 25, 26, and 27 of the BPTF gene (Figure 1C). Actinomycin D (Figure 1D) and RNase R treatment assays (Figure 1E) showed that circ-BPTF was more stable than was linear mBPTF. Moreover, random hexamer or oligo dT (18) primers were used separately in the reverse transcription assays with RNA from the T24 cell line as the template. Compared to random hexamer primers, the application of the oligo dT (18) primers resulted in significantly reduced relative expression of circ-BPTF (Figure 1F), though no significant difference was observed for the mBPTF group. This assay proved the absence of a poly (A) tail for circ-BPTF.

**Figure 2:**
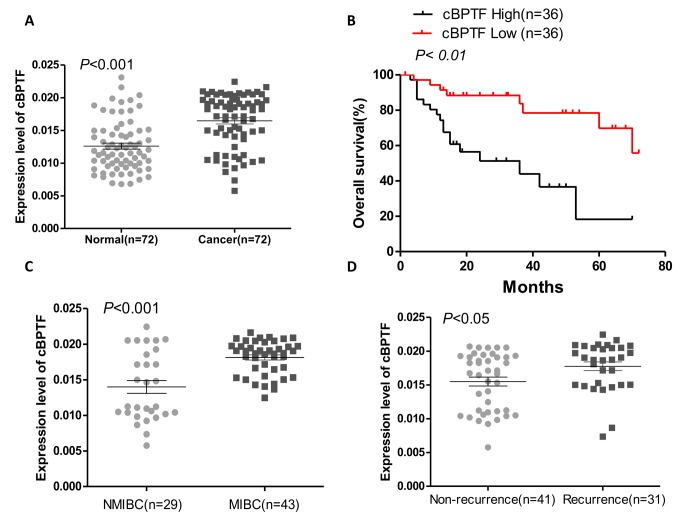
**Circ-BPTF expression and its clinical significance in BCa. **(**A)** The result of qPCR showed that circ-BPTF was up-regulated in BCa tissues compared to the adjacent normal tissues (P < 0.001). (**B**) Kaplan-Meier analysis demonstrated that patients with high circ-BPTF expression had poorer prognosis. Median expression of circ-BPTF in cancerous tissues was used as a cutoff value in survival analysis. (**C**) Expression of circ-BPTF in muscle invasive bladder cancer (MIBC) patients was notably higher than that in non-muscle invasive bladder cancer (NMIBC) patients (P<0.001). (**D**) Expression of circ-BPTF was significantly higher in patients with recurrence (P<0.05).

In addition, nuclear mass separation assay (Figure 1G) and FISH (Figure 1H) for circ-BPTF showed the circRNA to be predominantly located in the cytoplasm. Moreover, to validate expression of circ-BPTF in BCa cell lines, qPCR was performed. Circ-BPTF was significantly increased in BCa cell lines than in normal urothelial cell (SV-HUC-1) (Figure 1I). We also found that the expression level of circ-BPTF was higher in 72 pairs of human BCa tissues compared to corresponding adjacent normal tissues (Figure 2A). Collectively, these findings indicate that circ-BPTF is a circular, abundant and stable transcript with strongly increased expression in BCa.

### Circ-BPTF is related to tumor grade and recurrence and poor prognosis in BCa patients

To determine whether circ-BPTF correlates with the prognosis of BCa, patients were divided into two groups according to the expression level of circ-BPTF in BCa tissues. Based on Kaplan-Meier survival analysis, the group of patients with a high expression level of circ-BPTF had worse overall survival (OS) than in the group with low expression level of circ-BPTF (Figure 2B). Additionally, expression of circ-BPTF correlated with tumor stage and recurrence (Figure 2C, D), but not with other features, including metastasis and tumor size (Table 1). Overall, we concluded that higher expression of circ-BPTF leads to a worse prognosis for BCa.

**Table 1:** P<0.05 was considered significant.

Characteristics	Case	circ-BPTF expression		*P*
		Low (36)	High (36)	value
Age(years)				0.2196
< 65	26	10	16	
≥ 65	46	26	20	
Gender				0.7531
Male	60	31	29	
Female	12	5	7	
Pathology stage				0.5936
pTa-pT1	19	11	8	
pT2-pT4	53	25	28	
Histological grade				0.0043
Low	22	17	5	
High	50	19	31	
Tumor size(cm)				0.2384
< 3	36	21	15	
≥3	36	15	21	
Lymph nodes status				1
Negative	55	28	27	
Positive	17	8	9	

### Circ-BPTF promotes BCa cell progression in vitro and in vivo

**Figure 3:**
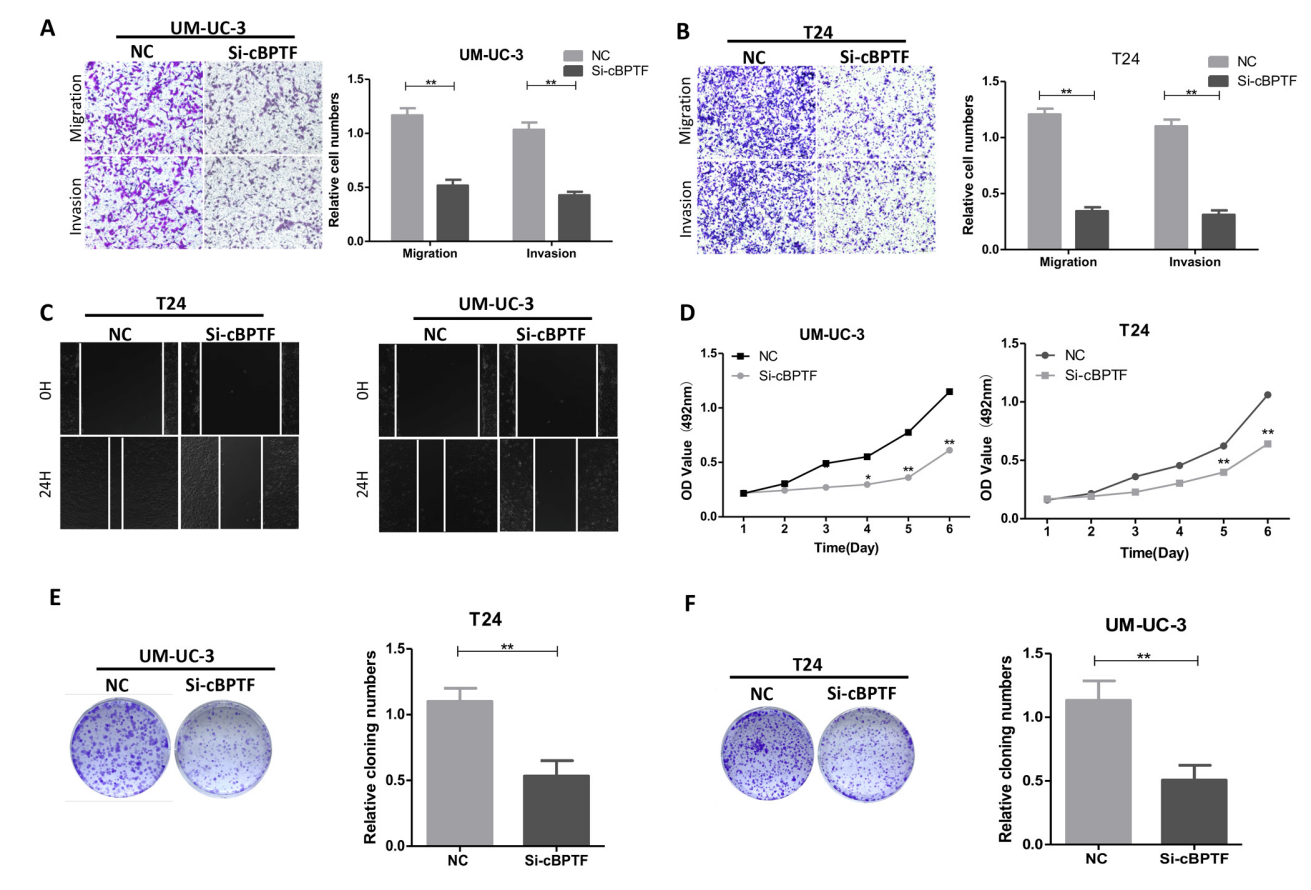
**Circ-BPTF promotes progression of BCa cells in vitro. **(**A, B **and** C**) Effects of circ-BPTF on cell migratory and invasive capabilities were assessed by transwell migration, Matrigel invasion and wound-healing assays. (**D-F**) MTS and clone-formation assays showed that the proliferative ability was decreased in T24 and UM-UC-3 cells transfected with si-circ-BPTF. Data indicate the means ± SEM. *P<0.05, **P<0.01.

**Figure 4:**
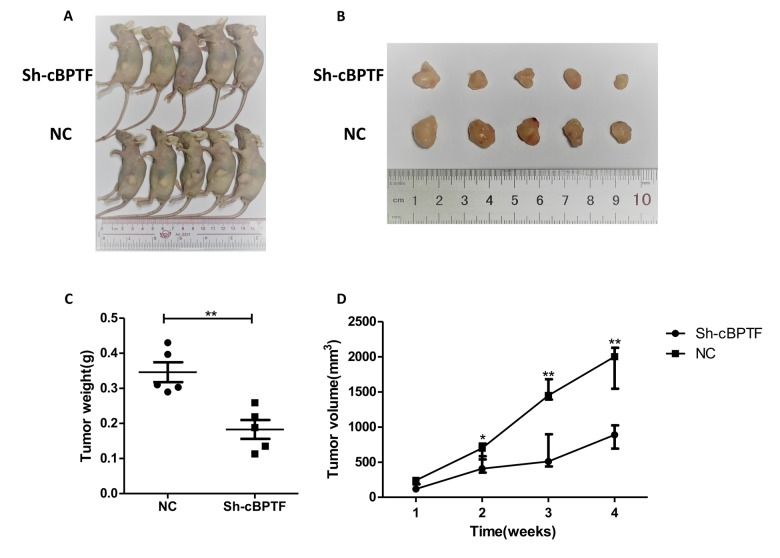
**Knocking down circ-BPTF suppresses the growth of BCa cells in vivo.** (**A)** Sh-circ-BPTF suppressed subcutaneous xenograft growth in vivo (n=5) (**B**) The Gross of subcutaneous xenograft tumors. (**C, D**) Analysis of tumor weight and volume of xenograft tumors. Data indicate means±SEM, *P<0.05, **P<0.01.

After knocking down circ-BPTF, UM-UC-3 and T24 cells were divided into si-circ-BPTF and negative control (NC) groups. Transfection had no obvious effect on expression of its parent gene BPTF (Figure S1A). The results of migration, invasion and wound-healing assays indicated that the migratory and invasive abilities of cells were inhibited in the silenced group (Figure 3A-C). MTS assay also revealed that the proliferative ability of the silenced group was suppressed compared to the NC group (Figure 3D). Accordingly, in clone-formation experiment, cells in the silenced group formed fewer colonies than those in the NC group (Figure 3E, F). In tumorous xenograft experiment, mice injected with cells that knocked down for circ-BPTF produced smaller and less massive tumors than mice injected with NC group cells (Figure 4A-D). Taken together, circ-BPTF promotes the progression of BCa in vitro and in vivo.

### miR-31-5p directly binds to circ-BPTF

**Figure 5:**
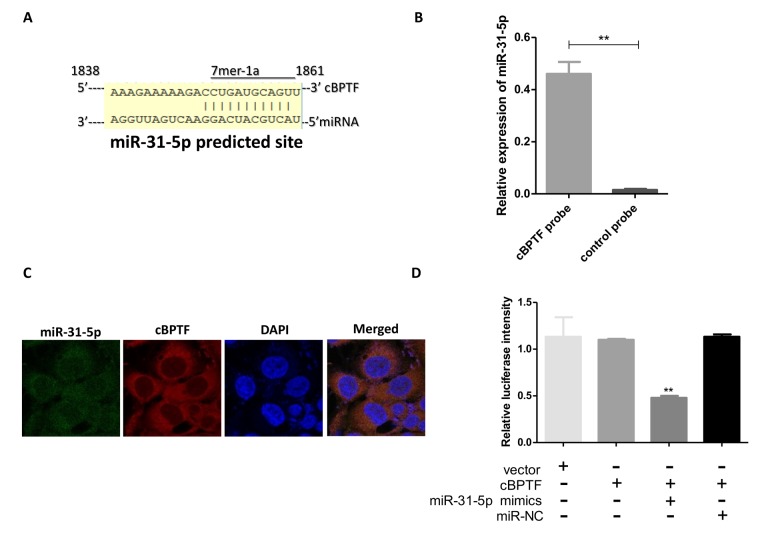
**miR-31-5p directly binds to circ-BPTF**. (**A**) Potential binding sites between circ-BPTF and miR-31-5p were predicted by CircInteractome. (**B**) miR-31-5p could be pulled down by the circ-BPTF probe. (**C**) FISH showed the co-localization between circ-BPTF and miR-31-5p. (**D**) Renilla luciferase activity in 293T cells co-transfected with miR-31-5p mimics and circ-BPTF reporter. Data indicate means±SEM. **P<0.01

The binding sites of circ-BPTF and miR-31-5p are displayed in Figure 5A, as revealed by bioinformatic database searches. To confirm binding between circ-BPTF and miR-31-5p, a biotin-coupled probe pull-down assay was performed. The results showed that miR-31-5p was significantly enriched in the pulled down material compared to the control group, indicating that miR-31-5p directly binds to circ-BPTF (Figure 5B). circ-BPTF was also found to be co-localized with miR-31-5p in the cytoplasm of BCa cells (Figure 5C). Furthermore, in a dual-luciferase reporter assay, co-transfection of miR-31-5p mimics and luciferase reporters containing circ-BPTF in 293T cells significantly reduced luciferase intensity (Figure 5D). These results suggest that miR-31-5p directly binds to circ-BPTF.

### miR-31-5p attenuates the function of circ-BPTF in BCa cells

**Figure 6:**
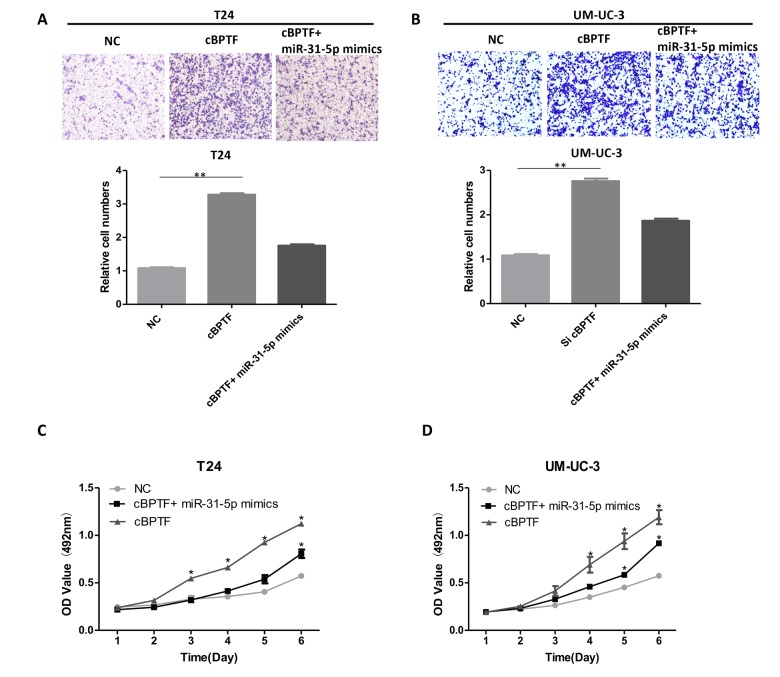
**Overexpression of miR-31-5p attenuates the oncogenic effects of circ-BPTF in BCa cells. **(**A**,** B**) The migratory ability enhanced by overexpression of circ-BPTF was attenuated after co-transfection with miR-31-5p mimics. (**C**,** D**) The proliferative ability enhanced by overexpression of circ-BPTF was also attenuated after co-transfection with miR-31-5p mimics. Data indicate means ± SEM. *P<0.05, **P<0.01.

To find out whether miR-31-5p could reverse the oncogenic effect of circ-BPTF, we co-transfected miR-31-5p mimics and circ-BPTF into BCa cells. As shown in Figure 6A-B, miR-31-5p mimics could partially attenuate the migrative ability of circ-BPTF in BCa cells. Besides, the proliferative ability of circ-BPTF was also reversed by co-transfection of miR-31-5p (Figure 6C, D). Taken together, these data demonstrate that miR-31-5p partially alleviates the oncogenic effects of circ-BPTF in BCa.

### Circ-BPTF promotes BCa progression through the miR-31-5p/RAB27A axis

**Figure 7:**
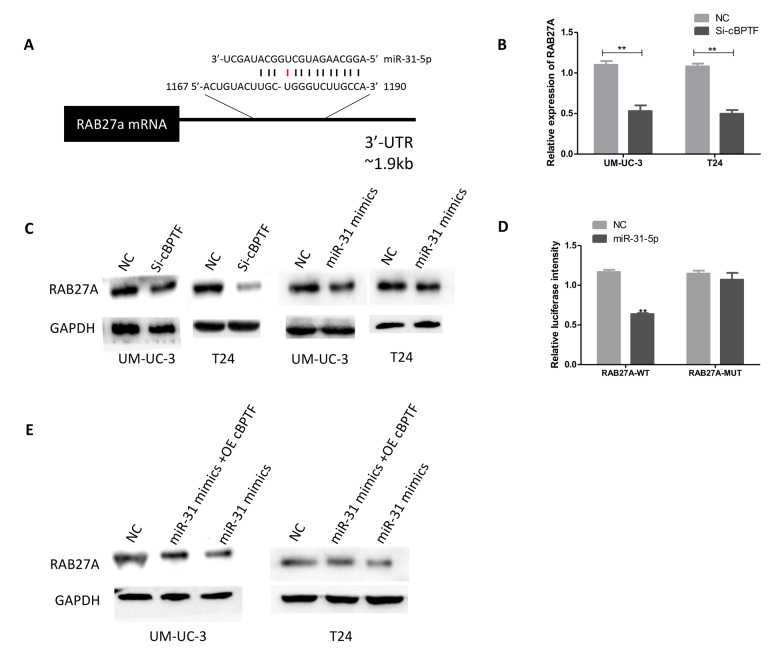
**Circ-BPTF promotes BCa proliferation and migration through the** **miR-31-5p/RAB27A axis.** (**A**) Schematic of predicted miR-31-5p binding sites in the 3’ UTR of RAB27A, with complementary pairs showed in black and mismatches showed in red. (**B**) Expression levels of RAB27A were detected following knockdown of circ-BPTF by qPCR**. (C**) Western blotting analysis of RAB27A in BCa cell lines upon knockdown of circ-BPTF and overexpression of miR-31-5p. GAPDH was used as a loading control. (**D**) miR31-5p decreases the luciferase activities of the wild-type RAB27A 3’ UTR reporter but not the luciferase activities of the mutant RAB27A 3’ UTR reporter. (**E**) Rescue experiment was performed to analyze RAB27A at protein level by western blotting. GAPDH was used as a loading control. **P<0.01.

We screened RAB27A as a target of miR-31-5p using bioinformatic analysis (Figure 7A) and then investigated whether circ-BPTF exerts its tumorigenic effect through the miR-31-5p/RAB27A axis. Knockdown of circ-BPTF decreased the expression of RAB27A at both mRNA and protein levels (Figure 7B, C). In addition, overexpression of miR-31-5p significantly reduced RAB27A levels (Figure 7C). Mechanistically, a dual-luciferase reporter assay showed that co-transfection of miR-31-5p mimics and a reporter containing the 3’-UTR ofRAB27A strongly reduced luciferase activity, however, no obvious effect on luciferase activity was found after co-transfection of miR-31-5p mimics and mutated vectors (Figure 7D). Furthermore, rescue experiment confirmed that miR-31-5p partially reversed the effect of circ-BPTF on RAB27A expression (Figure 7E). Overall, circ-BPTF promotes BCa progression partially through the miR-31-5p/RAB27B axis.

## DISCUSSION

In the present study, we confirmed that circ-BPTF serves as a miRNA sponge and promotes BCa progression at least partly through the miR-31-5p/RAB27A axis. We observed a significant increase in circ-BPTF expression in BCa tissues and cell lines compared with normal adjacent tissues and cells. Additionally, we found that overexpression of circ-BPTF correlated with poor survival, high tumor stage and tumor recurrence, suggesting that circ-BPTF exerts an oncogenic effect in BCa.

Only a few circRNAs have been reported in BCa thus far. For example, circ-ITCH can inhibit BCa progression by sponging miR-17/miR-224 and regulating p21 and PTEN expression [17]. Similarly, circHIPK3 inhibits the migration, invasion, and angiogenesis of BCa and suppresses BCa growth and metastasis by sponging miR-558 [21]. These studies demonstrate that circ-RNAs act as tumor suppressors, contributing to BCa progression. Conversely, there are few studies of oncogenic circ-RNAs in BCa. Indeed, our study is the first to report that circ-BPTF expression is increased in BCa, promoting progression of this cancer.

Functionally, our gain- and loss-of-function experiments showed that knocking down circ-BPTF significantly inhibited progression of BCa cells in vitro, which was verified by a xenograft experiment in vivo. Taken together, these experiments substantiate that circ-BPTF exerts oncogenic effects in BCa.

With regard to the mechanism, we selected miR-31-5p as a potential target miRNA of circ-BPTF via bioinformatic analysis. According to previous studies, the role of miR-31-5p in tumorigenesis is controversial. For instance, a previous study discovered that miR-31-5p promotes lung cancer progression by directly targeting FIH [23], and miR-31-5p can promote proliferation and aerobic glycolysis in breast cancer [22]. However, miR-31-5p has also been reported to enhance the sensitivity of BCa cells to chemotherapy [24] and target RAB27A to inhibit melanoma progression [26], which is consistent with our data. RAB27A is an oncogene belonging to the Rab family that is involved in protein transport and small GTPase-mediated signal transduction in multiple processes of tumorigenesis [25]. A previous study demonstrated that elevated levels of RAB27A expression promote BCa proliferation and chemoresistance [27]. Through bioinformatic analysis and dual-luciferase reporter assays, we confirmed that miR-31-5p is able to directly bind to the 3’UTR of RAB27A. Using western blot analysis, we identified that expression of RAB27A was down-regulated after silence of circ-BPTF and overexpression of miR-31-5p. Because RAB27A is known for its strong tumor-promoting effect in BCa progression, we hypothesized that the oncogenic effect that circ-BPTF exerts in BCa can be partially attributed to a circ-BPTF/miR-31-5p/RAB27A axis. Therefore, we conducted a rescue experiment showing that overexpression of circ-BPTF increased RAB27A expression and that this can be partially alleviated by miR-31-5p mimics in BCa cells.

In general, we reveal that circ-BPTF exerts an oncogenic function through a novel circ-BPTF/miR-31-5p/RAB27A axis in BCa. Because expression of circ-BPTF was found to be associated with BCa prognosis, this molecule possesses great potential in the diagnosis and treatment of BCa, and further investigation is warranted.

## MATERIALS AND METHODS

### Specimens

In total, 72 pairs of specimens of BCa tissues and adjacent noncancerous tissues were obtained via radical surgery of patients who were diagnosed with BCa and had undergone no pretreatment at Sun Yat-Sen Memorial Hospital, Sun Yat-Sen University between 2012 and 2017. All patients signed informed consent forms before inclusion in the study. Prior to RNA extraction, the fresh samples mentioned above were frozen in liquid nitrogen for 5 mins and stored at -80 °C. The protocol and written informed consent of the presented study were approved by the Ethics Committee of Sun Yat-Sen Memorial Hospital, Sun Yat-Sen University. Patients were followed up from the date of surgery to the date of BCa progression. Additionally, a cohort of 72 BCa patients with similar clinicopathological parameters was followed. The follow-up time ranged from 1 to 70 months. The follow-up interval began on the date of surgery and ended on the date of disease progression.

### Total RNA extraction and quantitative real-time PCR (qPCR)

Total RNA from frozen tissues and cells was homogenized and isolated using RNAiso Plus (Takara, Japan) according to the manufacturer’s instructions. cDNA was synthesized using the PrimeScript RT Reagent Kit (Takara, China) and microRNA First-Strand cDNA Synthesis Kit (Sangon Biotech, China). qPCR was performed with TB Green Premix Ex Taq (Takara, China) with the following program: 95 °C for 10 min and 40 cycles of 95 °C for 30 s, 55 °C for 30 s, and 72 °C for 30 s. Each reaction was performed in triplicate.

### Cell culture

Human BCa cell lines (UM-UC-3 and T24) purchased from ATCC (USA) were used in this study. UM-UC-3 cells were maintained in DMEM (Gibco, USA) with 10% fetal bovine serum (FBS), and T24 cells were maintained in 1640 medium (Gibco, USA) with 10% FBS. Cell lines were incubated at 37 °C in 5% CO_2_.

### Cell transfection

Before transfection, cells were seeded into 6-well plates at a density of 2×10^5^ cells per well and cultured to 60-70% confluence. For knockdown of circ-BPTF, a small interfering RNA (siRNA) oligonucleotide targeting circ-BPTF (si-circ-BPTF: AAGCUAGCAGGUACAGGUGTT) and negative control siRNA were purchased from GenePharma (Shanghai, China). siRNA transfections were performed with 75 nm siRNA and Lipofectamine RNAiMAX (Life Technologies, USA), as previously described [20]. The pLKO.1 TRC cloning vector (Addgene plasmid: 10878, China) was used to generate short hairpin RNAs (shRNAs) against circ-BPTF or the negative control. Lentivirus production and infection were conducted according to the manufacturer’s protocol.

### MTS assay

Transfected cells were seeded into 96-well plates at a density of 1000 cells per well, and 10 µl of 96® AQueous MTS Reagent Powder (Promega, Beijing, China) was added to each well. After 3 hours of incubation, we measured and recorded the absorbance of each well at 492 nm as the OD value every 24 hours for a total of 6 times; the manufacturer’s instructions were followed. Each group was analyzed in triplicate.

### Clone-formation assay

Transfected cells were seeded into 6-well plates at a density of 1000 cells per well. After culturing for 7 days, the cells were fixed with 4% paraformaldehyde and stained with 0.1% crystal violet. We then photographed the plates and counted the number of clones in each plate. Each group was analyzed 3 times.

### Migration and invasion assays

Migration and invasion assays were performed using transwell plates (Costar, USA). For the migration assay, transfected cells were suspended in serum-free medium at a concentration of approximately 400 cells per/μl and seeded into the upper chambers of each transwell; medium with 10% FBS as a chemoattractant was added to the bottom chambers. After incubation for 24 hours, the cells remaining in the top chambers were removed, and the cells in the lower chambers were fixed with 4% paraformaldehyde and stained with 0.1% crystal violet. Afterwards, the assays were quantified by counting the number of cells under a microscope at × 100 magnification (Olympus, Japan). The conditions for the invasion assay resembled those of the migration assay, except that Matrigel (BD Biosciences, USA) was coated onto each transwell before seeding, and the incubation time was extended to 48 hours.

### Xenograft experiments

Male BALB/c nude mice (4-6 weeks old, 18-22 g) were selected and randomly divided into two groups (sh and NC, 5 per group). Approximately 1×10^7^ UM-UC-3 cells transfected with sh-circ-BPTF or the negative control were subcutaneously injected into the backs of the nude mice. The width (W) and length (L) of tumors were measured every week and then integrated into one parameter as volume (V) using the formula V = (W^2^ × L)/2. Six weeks after the injection, the mice were euthanized, and the weights of the tumors were measured. The in vivo experiments were approved by the Institutional Animal Care and Use Committee of Sun Yat-Sen Memorial Hospital, Sun Yat-Sen University (Guangzhou, China).

### Biotin-coupled probe pull-down assay

To identify the downstream molecules of circ-BPTF, we conducted a biotin-coupled probe pull-down assay. Approximately 2×10^7^ UM-UC-3 and T24 cells were washed with ice-cold phosphate-buffered saline (PBS) and lysed in lysis buffer before being incubated with 3 μg of pull-down probes at 25 °C. A biotinylated circ-BPTF junction area-binding probe was used for the experimental group, and the oligonucleotide probe was used for the control group. After incubating with the probes for 2 hours, cell lysates were incubated with streptavidin magnetic beads (Life Technologies, USA) for another 4 hours to pull down the biotin-coupled RNA complex. The beads were washed5 times with lysis buffer, and the bound miRNAs in the pull-down complex were then extracted and analyzed.

### Fluorescence in situ hybridization (FISH)

Fluorescent probes targeting circ-BPTF (cy3-labeled) and miR-31-5p (cy5-labeled) were designed for in situ hybridization; 4,6-diamidino-2-phenylindole (DAPI) was used to stain nuclei. Hybridization was performed overnight following the manufacturer’s instructions (GenePharma, China). Afterwards, the stained cells were analyzed using a Zeiss LSM880 NLO (2 + 1 with BIG) confocal microscope system (Leica Microsystems, Germany).

### Dual-luciferase reporter assay

Plasmids carrying wild-type circ-BPTF sequences or wild-type RAB27A 3’UTR sequences plus miR-31-5p mimics were co-transferred for the experimental group; mutant sequences of the circ-BPTF or RAB27A 3’UTR plus miR-31-5p were co-transfected for the control group. The manufacturer’s protocol was followed. At 24 hours after transfection, the dual-luciferase reporter assay system (Promega, USA) was used to detect firefly and Renilla luciferase activities. Relative luciferase activity was calculated by comparing Renilla and firefly luciferase activities.

### Rescue experiments

BCa cells were divided into 3 groups. Circ-BPTF, circ-BPTF plus miR-31-5p mimics and a nonsense control were transfected into BCa cells using the method described above. After incubation with the MTS for 3 hours, the OD value of each well for each group was measured and recorded. Moreover, the expression of RAB27A was assessed by qPCR and western blotting.

### Statistical analysis

Statistical analyzes were conducted with SPSS 19.0 (IBM, SPSS, Chicago, IL, USA). Student’s t-tests (for quantitative data) or Chi-square tests (for categorical data) were used to analyze differences between two groups. Survival curves were generated using the Kaplan-Meier method, and the log-rank test was used to assess significance. A P-value < 0.05 indicated statistical significance.

## Supplementary Material

Supplementary Figure
