# Correction for: Circ‐BPTF promotes bladder cancer progression and recurrence through the miR‐31‐5p/RAB27A axis

**DOI:** 10.18632/aging.202597

**Published:** 2021-01-28

**Authors:** Junming Bi, Hongwei Liu, Zijian Cai, Wei Dong, Ning Jiang, Meihua Yang, Jian Huang, Tianxin Lin

**Affiliations:** 1 Department of Urology, Sun Yat‐Sen Memorial Hospital Sun Yat‐Sen University, Guangzhou, PR, China; 2 Guangdong Provincial Key Laboratory of Malignant Tumor Epigenetics and Gene Regulation, Sun Yat‐Sen Memorial Hospital Sun Yat‐Sen University, Guangzhou, PR, China

**Keywords:** correction

Original article: Aging. 2018; 10:1964–1976.  . https://doi.org/10.18632/aging.101520

**This article has been corrected:** The authors replaced in **Figures 3:** panel **3A**, where the UM-UC-3 migration/NC image was mistakably reused as the UM-UC-3 invasion/NC image and new image of “UM-UC-3 invasion/NC” from the same set of experiments was used for the new panel; and panel **3C**, where the 0h T24/NC image was mislabeled as the 0h UM-UC-3/NC and 0h T24/Si-cBPTF was mislabeled as the 0h UM-UC-3/ Si-cBPTF during the picture capturing. The new images of 0h UM-UC-3/NC and 0h UM-UC-3/ Si-cBPTF from the same set of experiments were used for the new panels.

The authors replaced in **Figures 7** panel **7C**, where the UM-UC-3(NC and miR-31 mimics) GAPDH image was mistakably reused as the T24(NC and miR-31 mimics) GAPDH image. The new panel **7C** contains new T24 images of GAPDH from the same set of experiments.

These alterations do not affect the results or conclusions of this work. The new **Figure 3** and **Figure 7** are presented below.

**Figure 3:**
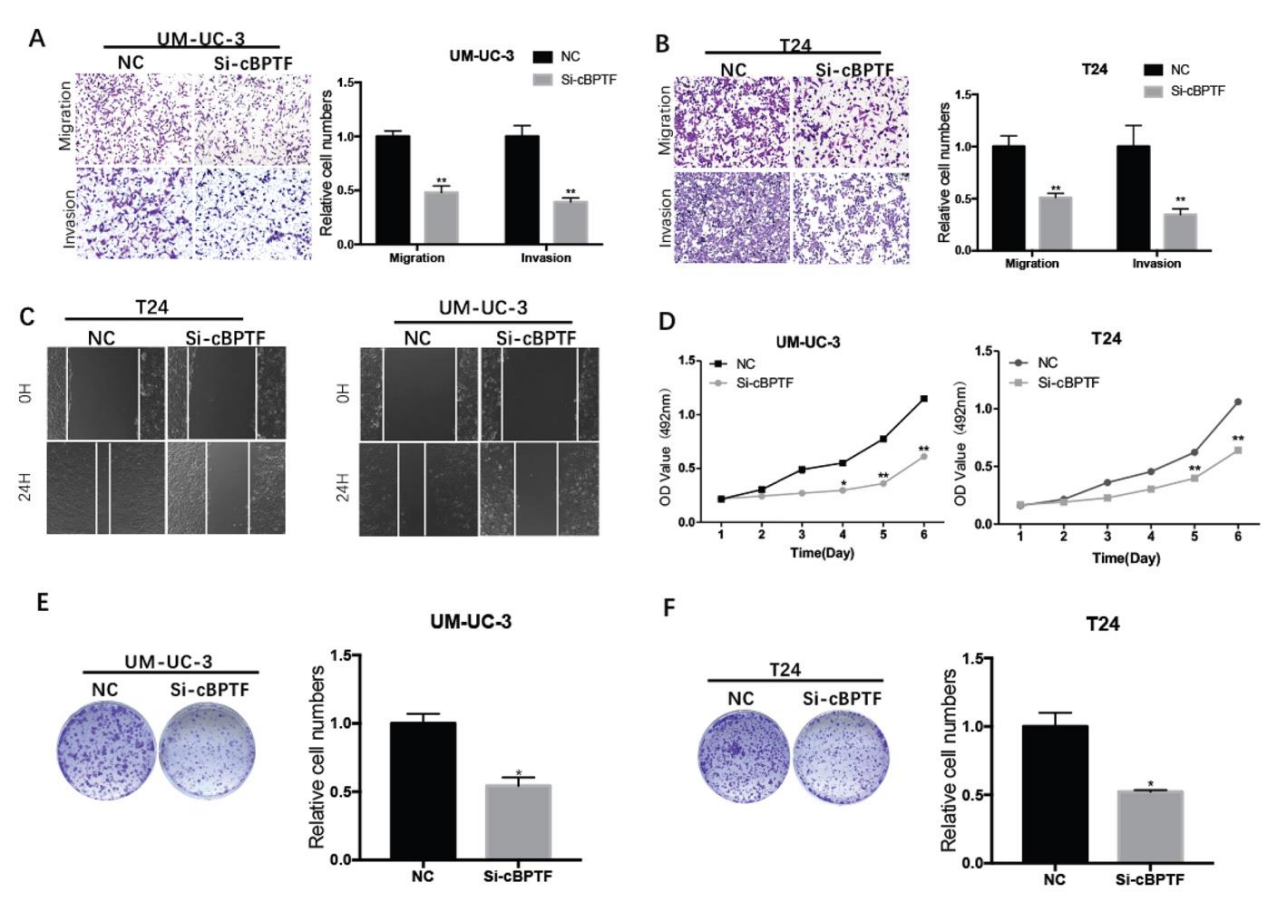
**Circ-BPTF promotes progression of BCa cells *in vitro*.** (**A, B** and **C**) Effects of circ-BPTF on cell migratory and invasive capabilities were assessed by transwell migration, Matrigel invasion and wound-healing assays. (**D-F**) MTS and clone-formation assays showed that the proliferative ability was decreased in T24 and UM-UC-3 cells transfected with si-circ-BPTF. Data indicate the means ± SEM. *P<0.05, **P<0.01.

**Figure 7:**
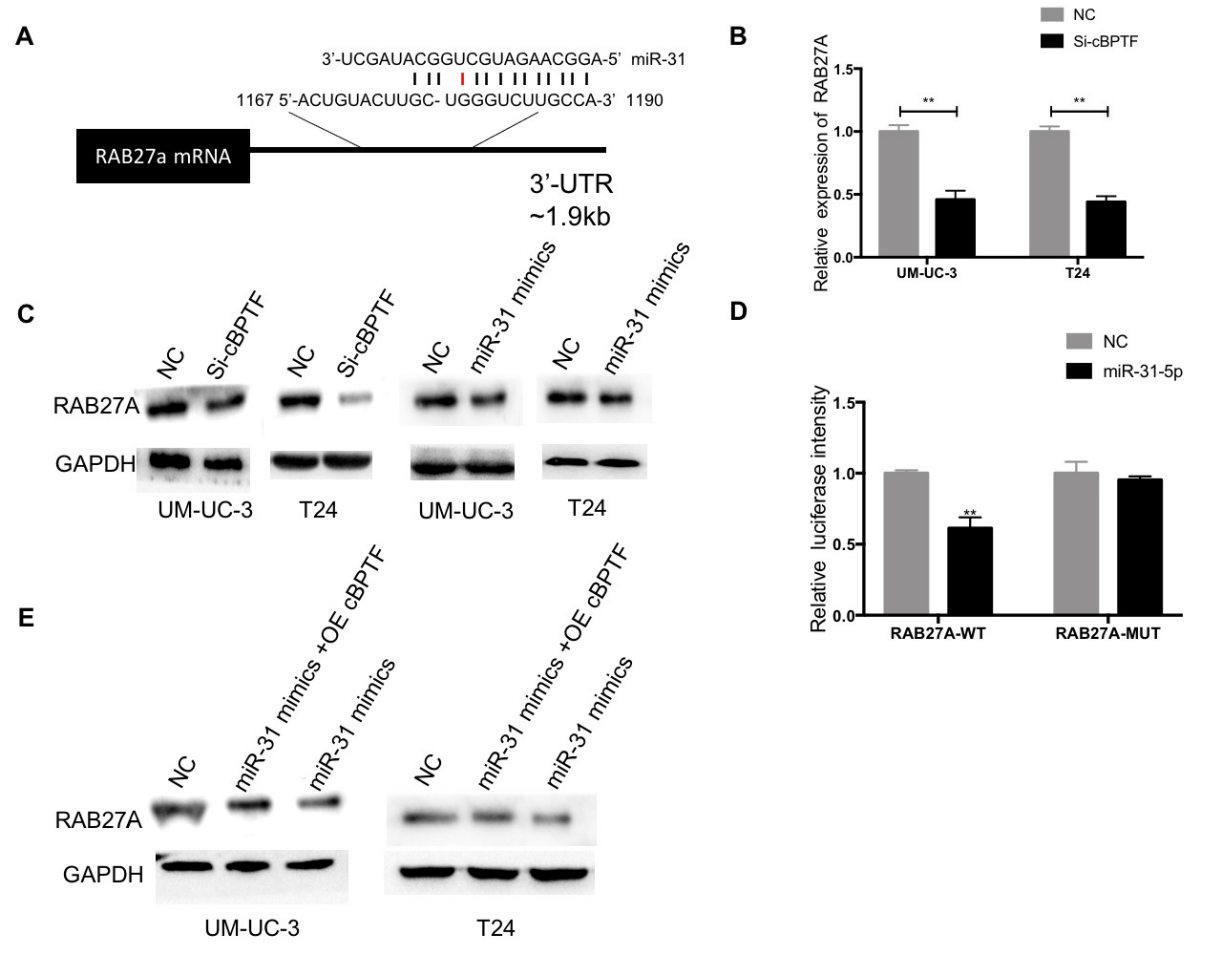
**Circ-BPTF promotes BCa proliferation and migration through themiR-31-5p/RAB27A axis.** (**A**) Schematic of predicted miR-31-5p binding sites in the 3’ UTR of RAB27A, with complementary pairs showed in black and mismatches showed in red. (**B**) Expression levels of RAB27A were detected following knockdown of circ-BPTF by qPCR. **(C**) Western blotting analysis of RAB27A in BCa cell lines upon knockdown of circ-BPTF and overexpression of miR-31-5p. GAPDH was used as a loading control. (**D**) miR31-5p decreases the luciferase activities of the wild-type RAB27A 3’ UTR reporter but not the luciferase activities of the mutant RAB27A 3’ UTR reporter. (**E**) Rescue experiment was performed to analyze RAB27A at protein level by western blotting. GAPDH was used as a loading control. **P<0.01.

