# Supplementary material for: Circ-BPTF promotes bladder cancer progression and recurrence through the miR-31-5p/RAB27A axis
Source: Aging (Albany NY). 2018 Aug 9;10(8):1964–76. doi: 10.18632/aging.101520 (PMC6128440; doi:10.18632/aging.101520)
Supplement: Supplementary Figure [file aging-10-101520-s001.pdf]

A

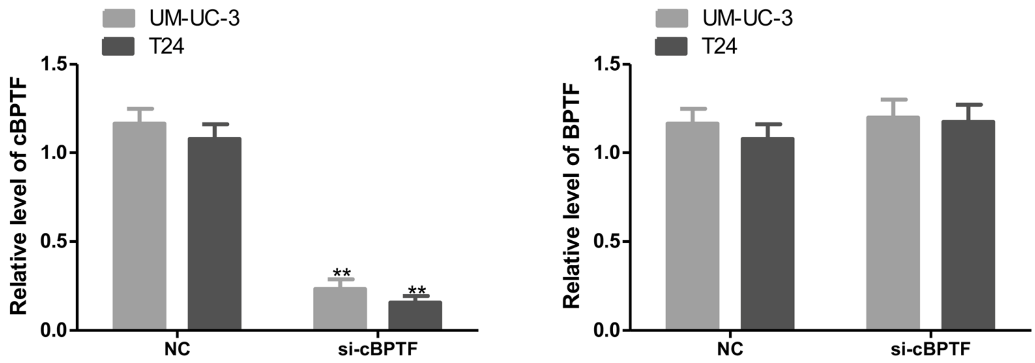

Figure S1. Knockdown of circ-BPTF had no significant influence on expression of linear BPTF. (A) Circ-BPTF knockdown only affected the circular transcript, with no significant influence on expression of linear BPTF.
